# Managing hyponatraemia in a patient with malignant melanoma: a case report

**DOI:** 10.4076/1757-1626-2-6899

**Published:** 2009-07-30

**Authors:** Siwan H Davies, Margred M Capel, Ilora Finlay

**Affiliations:** 1 St2, Core Medical Training, Velindre HospitalVelindre Road, Whitchurch, Cardiff, CF14 2TLUK; 2 The George Thomas Memorial TrustPark Road, Whitchurch, Cardiff, CF14 7BQUK; 3 Velindre HospitalVelindre Road, Whitchurch, Cardiff, CF14 2TLUK

## Abstract

We report the case of a 46-year-old male with a known diagnosis of metastatic malignant melanoma who presented with hyponatraemia. The report details the challenges we faced in identifying the cause of his hyponatraemia and in attempting to reverse his electrolyte disturbance.

As his clinical condition deteriorated the focus of our management needed to change; recognising that he was dying we implemented the Mental Capacity Act to make decisions in his best interest and ensure he achieved a symptom controlled and dignified death.

## Introduction

Hyponatraemia associated with malignancy is not uncommon; the symptoms and management are well documented [1]. However, the management of hyponatraemia in a patient with metastatic melanoma and adrenal metastases who has lost capacity to participate in treatment decisions is more complex. Balancing the aggressive management of his hyponatraemia against his clinical condition, and adapting this for end-of-life care, required tailoring the management to avoid burdening him with investigations and treatments. Using the Mental Capacity Act as a legal framework can aid decision making in this case and in similar cases.

## Case presentation

A 46-year-old Caucasian British male with metastatic malignant melanoma was admitted to our palliative care unit with worsening lethargy, generalized weakness, profuse sweating, abdominal pain and constipation.

Medication on admission included dexamethasone, commenced seven months previously for tiredness and general well being; the dose had recently been increased from 2 mg to 4 mg daily. Slow release morphine sulphate 90 mg 12 hourly and naproxen 500 mg b.d. for pain control, and warfarin following a pulmonary embolism and right iliac DVT diagnosed three months earlier. For relief of constipation he had taken six sachets of Movicol^®^ each day in the week prior to admission.

He had received his second cycle of carboplatin four weeks prior to this admission. Recent CT scan confirmed disease progression with tumour masses in the neck, superior mediastinum, left flank and the adrenal glands bilaterally (right 103 × 143 mm, left 122 × 164 mm). In addition, numerous subcutaneous tumour nodules, nodular liver metastases and significant retroperitoneal malignant lymphadenopathy were noted. CT imaging of the brain was unremarkable.

On examination he was euvolaemic without oedema. There was a firm, large, tender mass in the left upper quadrant. He was not confused and neurological examination was normal. There were numerous metastatic skin deposits of varying size visible on his face and neck.

His blood count, liver function, thyroid function and magnesium levels were all within normal reference ranges, but on admission his sodium was 106 mmol/L and potassium 5.9 mmol/L, urea 11.8 mmol/L and creatinine 104 mmol/L. Plasma osmolality was 242 mosmol/kg, bicarbonate 19 mmol/L, glucose 4.6 mmol/L and random cortisol 1 nmol/L. The initial urinary sodium was 79 mmol/L with a urinary osmolality of 596 mosmol/kg.

The blood results were surprisingly incongruous with the patient’s physical and mental state on this admission. The causes of hyponatraemia were considered with the intention of correcting his electrolyte imbalance in order to resolve his physical symptoms and restore his independent and active lifestyle.

The combination of hyponatraemia and hyperkalaemia with the evidence of significant bilateral adrenal tumours and long-standing dexamethasone therapy led us to the working diagnosis of hyponatraemia due to adrenal insufficiency secondary to the adrenal metastases. His electrolytes were documented within normal ranges two weeks prior to this admission. Other aetiologies considered included that the two recent cycles of carboplatin therapy had impaired his renal function leading to excess sodium loss or reduced sodium reabsorption, or that intense use of the osmotic laxative Movicol^®^ may have compounded his electrolyte imbalance.

The relevance of hyponatraemia as the underlying cause of his physical symptoms was discussed at length with the patient and his wife. Correction of his electrolyte imbalance was agreed upon following advice from the clinical biochemist.

Initial management strategies included commencing fludrocortisone, restricting his fluid intake and discontinuing his Movicol^®^. Over the following days his sodium remained between 105-108 mmol/L and his clinical condition remained stable; whilst he was mostly confined to bed and chair he remained conscious without seizures.

On day five of his admission he complained of sudden onset left loin pain, much more severe than his usual background pain, and became hypotensive and tachycardic. It was hypothesized that he had haemorrhaged into his adrenal tumour as his INR was 3.2 and Hb 9.7 g/dl having fallen from 11.4 g/dl on admission. The patient participated in discussions regarding his management and declined a more aggressive approach including further radiological investigations. He was commenced on a continuous subcutaneous infusion of 100 mg diamorphine and 10 mg midazolam over 24 hours to manage his pain. The dose was titrated against his background opioid use and additional requirements. Ketamine was used for additional analgesia and an opioid-sparing measure. Hydrocortisone was prescribed in place of dexamethasone and his warfarin was discontinued.

On day seven the patient was stable, his urinary sodium had dropped from 79 mmol/L to 5 mmol/L but the plasma sodium remained at 106 mmol/L. The plasma sodium increased to 109 mmol/L by day nine and to 112 mmol/L by the twelfth day of admission. Unfortunately, as the patient’s biochemistry improved his clinical condition then deteriorated. He became confused, aggressive, agitated and difficult to nurse.

As the patient had become unable to participate in decision-making, the Mental Capacity Act 2005 guidance - to involve and consider information provided by people who are best placed to express the prior thoughts and wishes of the patient - was followed. Prior to this episode he had discussed at length with his wife the extent to which he wanted interventions and treatments, saying he would decline further investigations and treatments including intravenous medication in the event of clinical deterioration, fully understanding the implications of this decision.

The clinical situation had evolved from admission. The patient was entering his terminal phase for a combination of reasons. Pursuing resolution of his electrolyte imbalance would no longer be in his best interests in this context nor would it arrest the dying process. It would contradict his wishes as reported by his wife and the views he had expressed to the medical team when he was capable of expressing his own autonomous informed opinions.

Analgesia and sedation were continued via the syringe driver and intravenous medication, including the hydrocortisone, ceased when peripheral access was lost. Central access was not attempted in light of the patient’s prior wishes. The patient died with his wife present fourteen days after admission.

## Discussion

The management of this patient presented us with a number of challenges, both clinical and ethical with some questions remaining unanswered. Hyponatraemia may have accounted for the majority of his symptoms (nausea, weakness, confusion in his last days), but on admission he appeared surprisingly well and was not confused despite his serum sodium of 106 mmol/L.

The interpretation and management of hyponatraemia is complex and not based on plasma sodium alone. Fundamental to its assessment is a measure of the patient’s hydration status. Identification and correction of the underlying cause is essential if the electrolyte imbalance is to be reversed. In broad terms hyponatraemia can be divided into hypovolaemic and eu/hypervolaemic hyponatraemia, both types can be further subdivided according to the urinary sodium concentration (less than or more than 20 mmol/L). A thorough history of drug use, fluid loss and examination of circulatory volume status and measurement of urinary sodium concentration is imperative in determining the cause and subsequently devising an appropriate management strategy.

The aetiology of his hyponatraemia remained unproven and was likely to be multifactorial. Several potential causes were identified from the history. No consent to postmortem was obtained.

Nephrotoxic insults included both the NSAID he had used in the weeks prior to admission and the recent cycles of carboplatin. This may have contributed to excessive sodium loss from the proximal tubule of the kidneys and some failure of reabsorption. The NSAID was discontinued on admission; the improvement in urea and creatinine and subsequent improvement in plasma sodium following the dramatic drop in urinary sodium supports this hypothesis. The prolonged high volume consumption of Movicol^®^ may have contributed to his hyponatraemia. There are published case-reports and data from the Medicines Control Agency documenting hyponatraemia secondary to the use of osmotic laxatives such as Movicol^®^ [2].

A strong candidate for the cause of his hyponatraemia, and our working diagnosis, on admission was adrenal insufficiency secondary to metastatic destruction of his adrenal glands, suppression of his pituitary-adrenal axis following long term treatment with dexamethasone and possibly reduced dexamethasone absorption as a result of vomiting and constipation. The random cortisol of 1 nmol/L supports this theory although a Synacthen^®^ test was not performed following advice from our biochemist with respect to meaningful interpretation. The patient was prescribed adrenal replacement therapy initially with oral dexamethasone (low mineralcorticoid activity) and fludrocortisone, respecting his preference for oral medication.

In practice it is likely that a combination of all these factors contributed to his hyponatraemia, and the symptoms that he experienced. The attempt to reverse his hyponatraemia was undertaken on the principle of beneficence based on his physical condition on admission being incongruous with his electrolytes and extent of his metastatic disease. It was his preference in light of his extensive disease to remain on the inpatient unit and not transfer for the more aggressive management within the hospital. He was fully informed regarding the anticipated outcome of the decisions he made to limit his treatment and investigations, yet he and his wife remained extremely grateful for the effort and work performed by the team to try and reverse his hyponatraemia and in communication, keeping them informed and involved in decision making.

His treatment and investigations were limited by the place of care; the complete reversal from 106 mmol/L was always going to be a difficult challenge, and one that we unfortunately failed to overcome on the inpatient palliative care unit. Following treatment for 10 days with little apparent improvement in his serum sodium concentration he became too unwell to be able to justify ethically regular blood tests. It is possible that his sodium continued to improve until death; on the other hand it may have plummeted following cessation of hydrocortisone when intravenous access was lost and hastened his demise. Application of the Mental Capacity Act guidance enabled his wife to feel she continued to participate in decision-making once the patient had lost capacity and that the clinical team adhered to her husband’s wishes as she perceived them.

There was considerable debate within the clinical team regarding the management of this case particularly when peripheral access was lost and central line placement was potentially required. The debate included whether the patient was dying with or as a consequence of his hyponatraemia and the extent to which this should be pursued. Following lengthy discussions, the whole team were confident that the eventual management decisions made were in the patient’s best interests as appraised by these basic ethical values.

## Conclusion

Adoption of an ethical framework in the management of medical cases and maintaining an open dialogue with the patient and their carers can facilitate decision making in such complex cases. Palliative care works within the confines of the healthcare system and the constraints of individual patient’s circumstances, yet management can be tailored to the individual, as this case illustrates.

## Abbreviations

CT: computerized tomography
DVT: Deep vein thrombosis
NSAID: Non steroidal anti-inflammatory drug
